# Key community eye health messages

**Published:** 2018-06-03

## If you see something white inside a child's eye, seek help

**Figure:**
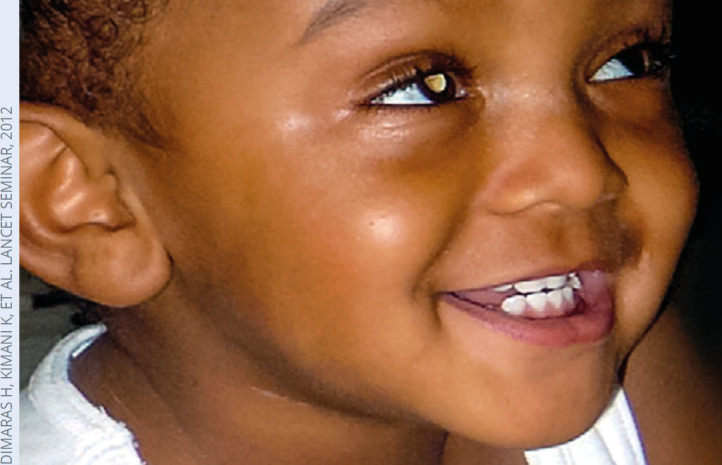

- This is easier to see in the early morning or late evening, or by a dim light
- Vision may not be affected
- Be determined. Do not let any one turn you away until a specialist eye doctor has looked inside the child's eyes
- Health workers: believe the parents/carers and refer to a specialist - it is an emergency

## Test the red reflex if you are able to

**Figure:**
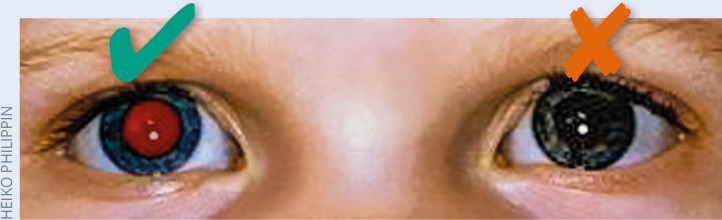
Left eye: the absence of a red reflex is abnormal and could indicate a serious condition. The reflex in the right eye is normal

**Figure:**
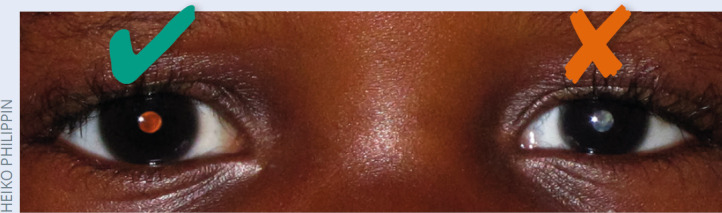
Left eye: The wrong colour reflex (here white). The child has a cataract in the left eye and needs urgent treatment to avoid permanent vision loss

- The reflex should be the same in both eyes
- Check the red reflex in a relative's eye for comparison if you are unsure
- An abnormal, or absent, red reflex in one or both eyes is serious and may indicate cataract or retinoblastoma
- An absent red reflex is also abnormal
- An abnormal red reflex is an emergency. Refer the child to a specialist urgently
- If you have any doubts, refer to a specialist who can examine the back of the eye

## Enucleation saves lives. With well fitted prostheses, children can have an attractive outcome

**Figure:**
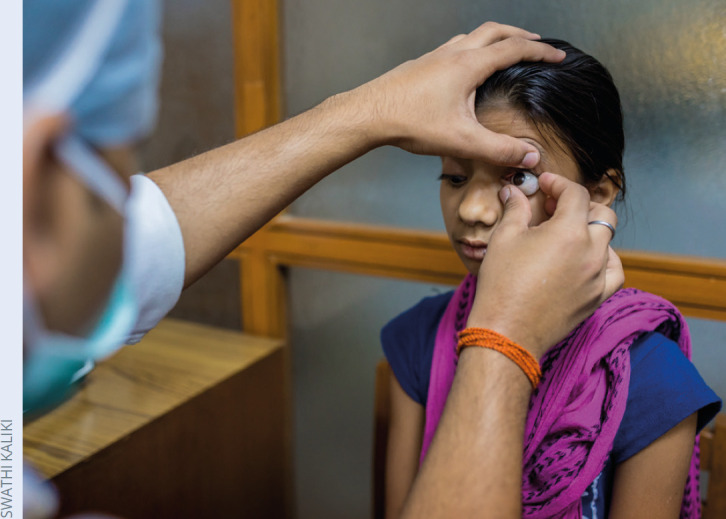
After healing, a prosthesis is fitted in the left eye

**Figure:**
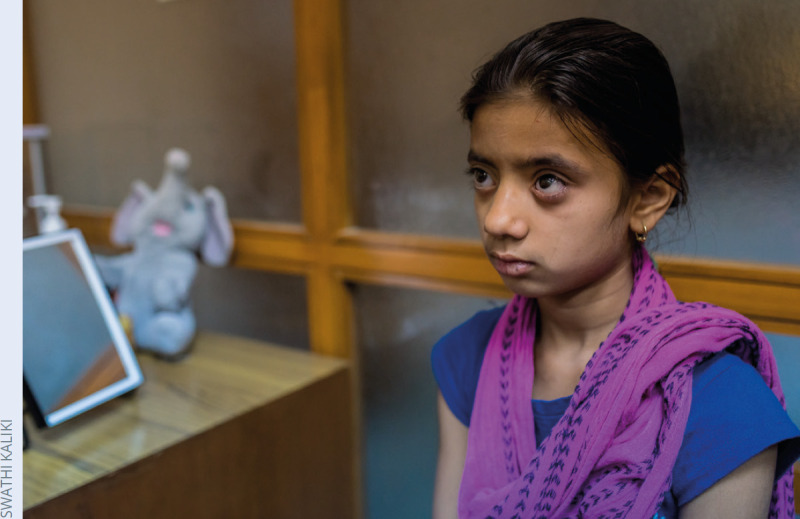
There is very little difference between the two eyes, allowing this girl to have a normal life

